# Fragile DNA contributes to repeated evolution

**DOI:** 10.1186/s13059-019-1655-x

**Published:** 2019-02-21

**Authors:** Claudius F. Kratochwil, Axel Meyer

**Affiliations:** 1 Department of Biology, University of Konstanz, Konstanz, Germany; 2 Zukunftskolleg, University of Konstanz, Konstanz, Germany

## Abstract

Sequence features that affect DNA fragility might facilitate fast, repeated evolution by elevating mutation rates at genomic hotspots.

While nature constantly continues to amaze with its astonishing diversity of life forms, sometimes, even evolution repeats itself. These “*evolutionary déjà-vus*” as Jonathan Losos calls them in his recent book “Improbable Destinies” [1] suggest that nature has found the same solutions to a similar ecological problem. In only a handful of examples do we know the genetic bases of (repeated) adaptations, and for many of these the underlying causal genes are shared. The question that then arises, are there any special features of these evolutionary genomic hotspots that explains their frequent, almost predictable involvement in adaptive transitions and repeated evolution [2]? A recent publication in *Science* propose that DNA fragility itself at such an evolutionary genomic hotspot might greatly facilitate repeated evolution [3].

## Repeated colonization of a new world

**Fig. 1:**
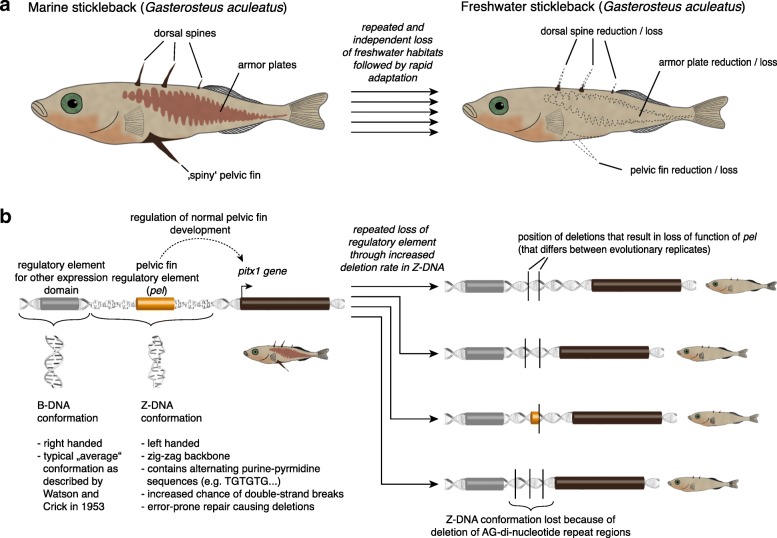
Molecular mechanisms of repeated pelvic fin loss in sticklebacks. **a** Three-spined sticklebacks (*Gasterosteus aculeatus*) repeatedly colonized postglacial freshwater habitats. The adaptations in these independent populations are remarkably similar. **b** One common adaptation is the loss of the paired spiny pelvic fins. This loss is caused by the repeated deletion of a pelvic fin specific regulatory element that drives expression of *pitx1*, a crucial transcription factor for pelvic fin development. The exact deletions differ between freshwater populations and as Xie et al. show [3] are facilitated by sequence features in the genomic region that result in a non-canonical DNA conformation (Z-DNA) that causes double-strand breaks repaired by the more error-prone non-homologous end-joining repair

When the last Ice Age ended about 10,000 years ago and glaciers started to melt, new streams and lakes formed in the northern hemisphere. Among the beneficiaries of this climatic change was a normally ocean-dwelling fish species, the three-spined stickleback (*Gasterosteus aculeatus;* Fig. 1a) that successfully colonized the newly forming freshwater habitats in areas that used to be covered by ice [4]. This new environment posed novel challenges for sticklebacks, including different predators, food sources and lack of salinity. Interestingly, different populations across the species’ range responded in astonishingly similar ways to the new freshwater lifestyle. These geographically distinct populations lost their armored plates and defensive spines, and also evolved more pronounced elongated or deep body shapes, as well as different reproductive and foraging behaviors [4, 5] (Fig. 1a). Defying conventional evolutionary expectations, these repeated adaptive responses evolved within often extremely short evolutionary timespans of less than a dozen generations, raising the question of how such dramatic and in particular repeated adaptations can occur so rapidly [4].

## A textbook example of repeated adaptation: Parallel losses of spiny fins

One of the most characteristic adaptations of sticklebacks to their new freshwater environments is the loss of the spiny structures characteristic of marine sticklebacks. This transition occurred independently many times [5] and includes the loss of needle-like pelvic fins on their ventral surface (Fig. 1a). The reduction of these skeletal elements is interpreted as an adaptation against invertebrate predators, more prevalent in freshwater habitats than marine, that could otherwise grab young stickleback by these structures. The genetic basis for this bone loss is largely driven by recurrent deletions of a regulatory region of *pitx1*, a pivotal gene for the development of the pelvic fin [5]. Interestingly, in spite of the extremely young evolutionary history of adaptation to fresh water, these deletions differ in size and are therefore considered to have been lost independently at the same position, bringing about the same phenotypic result through the loss of the pelvic fin regulatory element (*pel*) that is located within these deletions [5] (Fig. 1b). But how is it possible that the same evolutionary path was independently taken so often, caused by novel and seemingly independent mutations within a such an extremely short span of evolutionary time [3, 5]? This conundrum has now been solved in a recent paper [3] from the laboratory of David Kingsley.

## The pelvic fin *pitx1* enhancer: A story of repeated break-ups

What is special about the genomic region that encompasses the *pel* regulatory element? As it turns out, this genomic region is particularly rich in repeats, especially in TG-di-nucleotides [3] and this has far-reaching consequences. DNA mostly forms the right-handed double helix (B-DNA) as first shown by Watson and Crick [6] (Fig. 1b). However, several factors are able to trigger alternative tertiary DNA structures. Alternating purine–pyrimidine sequences often lead to the formation of the left-handed zigzag shaped Z-DNA [6] (Fig. 1b). To experimentally test the presence of the alternative structure Xie et al. used 2-D electrophoresis to screen for mobility shifts caused by the Z-DNA confirmation. In this in vitro assay, shifts could be only found using the marine sticklebacks *pel* sequence, but not the freshwater stickleback *pel* sequence where the Z-DNA conformation inducing repeats are naturally deleted [3] (Fig. 1b).

But what are the functional consequences of the non-canonical Z-DNA tertiary structure and can they explain the fast evolution occurring at the *pel* region [3]? Previous work in mammalian cells suggested a link between Z-DNA and elevated mutation and deletion rates resulting from a higher occurrence of DNA double-stranded breaks and a more error-prone microhomology-mediated end-joining repair [7]. To test this hypothesis Xie and co-authors used yeast artificial chromosomes (YACs) in which they inserted the *pel* region of different stickleback populations. The differences were striking: YACs with the *pel* sequence from marine populations tend to break 25–50 times more often than *pel* from freshwater sticklebacks that have lost their pelvic fins [3]. But it is not the sequence alone, and orientation of the sequence also matters. DNA replication direction influences the fragility of the DNA stretch, as shown by addition of a second replication origin after the *pel* sequence that drastically reduces the vulnerability for double-stand breaks. Using an elegant approach combining cell-sorting and genome resequencing, they could also confirm in vivo that the replication starts in front and not after the *pel* sequence. The authors further demonstrate that this effect is mainly mediated by the Z-DNA inducing TG-dinucleotide repeats and that this effect can be confirmed in mammalian cells [3]. Crispr-Cas9 mutants of the sequence provide further evidence that the deletion can indeed explain pelvic fin reduction. The large deletions observed in the Crispr-Cas9 mutants support the hypothesis of the impaired DNA repair mechanisms at this locus [3].

## Is fragile DNA more prone to contribute to adaptive evolution?

What are the implications of a such a mechanism explaining the rapid and repeated occurrence of variation at the *pitx1* locus that facilitates the repeated loss of the spiny pelvic fins during evolution? Locally elevated mutation rates might facilitate the accumulation of potentially beneficial mutations within the *pel* region of freshwater populations [3, 8]. With typical mutation rates this would be very unlikely to occur, as the authors demonstrate by population genetic modeling [3]. However, several factors have to be considered regarding the general importance of mutational biases in distinct genomic regions for repeated adaptive evolution.

Sticklebacks are a prominent example for the significance of standing genetic variation, where selection does not act on novel mutations but mutations that are already present at low frequency within the source population and then increase rapidly in frequency in the derived freshwater populations [4]. But, under what conditions (e.g. population size and selection history, genomic architecture of the trait, underlying molecular mechanism) are fragile genomic regions expected to be able to affect adaptive trait evolution compared to standing genetic variation? In case of the stickleback pelvic fin loss, we are dealing with the loss of a trait that can be acquired by different mutations at a single, Mendelian locus [5]. What would happen if we dealt with a polygenic trait? Or could the evolution of a novel regulatory region (whereby much more specific mutations must occur than for the deletion of a regulatory element) be facilitated by a fragile DNA region?

Another interesting issue pertains to the stability of the region of fragile DNA in marine populations: how frequently do novel mutations arise at this locus in the marine source populations? One might expect that there would be a cost for increased fragility in an important regulatory sequence (at least for marine sticklebacks) that confers an adaptation in the form of protective pelvic spines. As the trait is recessive, one would assume that deletions accumulate – despite stabilizing selection – at low frequency within marine stickleback populations. And why did no alleles arise that lack the repeats but maintain regulatory activity? Are there additional constraints that slow down this process?

Many open questions remain. DNA conformation is known not only to be affected by the primary DNA sequence, but also by the cellular environment and DNA binding proteins [6]. Do minor ionic or hydration differences affect the formation of Z-DNA and thereby reduce the *pel* fragility in marine stickleback? Are there additional genetic factors that reduce the vulnerability of the *pel* sequence in the marine environment? Maybe those are far-fetched, but not impossible mechanisms that contribute to the maintenance of the characteristic repeat-rich region in *pel*. The various roles and effects of Z-DNA are certainly controversial yet also include transcriptional regulation [9]. It might be therefore even possible that the TG-di-nucleotide repeats modulate the regulatory activity of *pitx1* itself and are therefore maintained by stabilizing selection.

## Concluding remarks

In the last decade great progress has been made in finding the genetic bases of the repeatedly evolved adaptations [2]. The study by Xie et al. demonstrates that we sometimes need to dig much deeper to reveal the molecular mechanisms that evolution has come up with in the struggle for life [3]. Natural experiments, such as the repeated invasion of post-glacial freshwater habitats by sticklebacks, or the parallel colonization of crater lakes by cichlid fish [10] provide rare opportunities to investigate the genomic and molecular basis of parallel adaptations that evolved extremely rapidly and repeatedly. Several recent studies supported the notion that evolution, at least at short time spans, can result in quite predictable, seemingly even deterministic outcomes [2]. While natural selection, acting at the phenotype, might appear predictable, Xie et al. newly discovered molecular mechanisms highlights that there is a suite of ways to reach the same outcome. In the future the search for the mechanistic bases of evolutionary change will also have to include less obvious causes such as DNA structure, sequence composition, chromatin state, topologically associating domains (TADs), and nuclear positioning. Apparently, a whole new level of complexity remains to be discovered.

## Abbreviations

*pel*: Pelvic fin specific regulatory element of Pitx1
*TAD*: topologically associating domain
YAC: Yeast artificial chromosomes
